# Research on the Mechanical Properties and Microstructural Evolution of Al-Si Alloy for Automotive Rear Floors Based on Simulation-Assisted Casting

**DOI:** 10.3390/ma18092143

**Published:** 2025-05-06

**Authors:** Liang Gao, Qiang Wang, Qin Yang, Wenjun Liu, Bin Jiang, Yongrui Qin, Haoming Chen, Sha Lan

**Affiliations:** 1 Chongqing Changan Automobile Co., Ltd., No. 260, Jianxindong Road, Jiangbei District, Chongqing 400030, China; gaoliang@changan.com.cn (L.G.); qinyr@changan.com.cn (Y.Q.); chenhm1@changan.com.cn (H.C.); lansha1@changan.com.cn (S.L.); 2 College of Materials Science and Engineering, Chongqing University of Technology, No. 69, Hongguang Road, Banan District, Chongqing 400054, China; wangqiang@stu.cqut.edu.cn (Q.W.); wjliu@cqut.edu.cn (W.L.); 3 College of Materials Science and Engineering, Chongqing University, No. 174, Shazheng Street, Shapingba District, Chongqing 400044, China

**Keywords:** Al-Si alloy, filling simulation, microstructural evolution, mechanical property

## Abstract

Al-Si alloys are essential in manufacturing automotive body structural components due to their superior casting properties, high specific strength, and excellent corrosion resistance. The microstructural evolution and mechanical properties of Al-Si alloy used in rear floors were systematically investigated based on a casting simulation. The results indicate that the alloy microstructure consists of α-Al, an Al-Si eutectic, and the Al_15_(Mn,Fe)_3_Si_2_ intermetallic phases. The accumulation of Al_15_(Mn, Fe)_3_Si_2_ intermetallic compounds increases toward the end of the filling process, leading to a reduction in mechanical properties. The optimal filling distance of the alloy ranges from 210 mm to 450 mm, while the optimal thickness ranges from 3.36 mm to 4.14 mm. With a filling distance and thickness increase, the yield strength, tensile strength, and elongation of the alloy initially increase and then decrease. The optimal properties are achieved when the filling distance is 210 mm and the thickness is 4.14 mm, with a yield strength of 122.35 MPa, a tensile strength of 258.43 MPa, and an elongation of 11.60%. At the same filling distance, near the gate position, when the thickness increases from 4.1 mm to 5.3 mm, the alloy’s tensile strength and elongation decrease. However, at positions farther from the gate, when the thickness increases from 2.94 mm to 4.93 mm, both the tensile strength and elongation of the alloy increase. This study provides a theoretical basis for the process design of large integrated die-casting components for new energy vehicles and supports the development of a high-strength ductile Al-Si alloy material system.

## 1. Introduction

To achieve dual-carbon goals, the automotive industry is rapidly advancing toward electrification and intelligent technologies. Light-weighting has become a crucial technological strategy for enhancing the driving range and dynamic performance of new energy vehicles [1,2]. Al-Si alloys are essential in manufacturing automotive body structural components due to their superior casting properties, high specific strength, and excellent corrosion resistance [3,4,5]. Large-size die-cast aluminum alloy structural components are gradually replacing traditional stamped-welded body structures. As a key load-bearing component, the vehicle’s rear floor significantly enhances overall performance through innovations in its manufacturing process [6]. Research indicates that the stiffness characteristics of the rear floor directly affect the noise, vibration, and harshness (NVH) performance and crash safety of the entire vehicle. The structural integrity of the rear floor contributes over 18% to the vehicle’s overall torsional rigidity [7]. Traditionally, the manufacturing of automotive rear floors involves stamping multiple steel sheets, followed by resistance welding to join them [8]. This approach presents several challenges, including a large number of components, a high density of connection points, low material utilization, extended production cycles, and difficulties in integrating complex structures [9].

Integrated die-casting technology enables the overall shaping of components using ultra-large die-casting machines, offering significant advantages in the manufacturing of automotive rear floors [10]. Design studies indicate that this technology can reduce the weight of the rear floor by 7% [6]. Zhang et al. [11] demonstrated that an integrated die-casting production of the rear wheel arch and seat crossbeam structure can reduce the weight of the welded area by 2.7 kg, while improving the body’s comfort stiffness by 6%. However, this technology still faces challenges in process control. During die-casting, the rapid movement of the molten material and the fast cooling rate often lead to defects, such as gas porosity and shrinkage [12]. These defects not only reduce the mechanical properties of the castings but may also result in casting failure [13]. Zhang et al. [14] observed that variations in injection speed transition positions can cause fluctuations in mechanical properties of up to 13%, and changes in porosity near the chamber significantly affect the reliability of the casting. The application of numerical simulation techniques in the casting field can effectively address the aforementioned issues, by simulating changes in the flow field, temperature field, and microstructure during the casting process, as well as predicting the potential defect locations. This approach helps optimize mold designs, shorten the design cycle, reduce production costs, and ultimately enhance the economic benefits of enterprises [15].

Current research primarily focuses on optimizing production process parameters, enhancing material properties. Lu et al. [16] determined the optimal process parameter combination for producing engine end caps from ADC12 alloy through orthogonal experiments: a pouring temperature of 680 °C, an injection speed of 4.0 m/s, and a mold preheating temperature of 220 °C. The product quality has been significantly improved. Li et al. [17] developed a local extrusion compensation technique for thick-walled regions of aluminum alloy rear end caps made from ADC12 alloy. Numerical simulation results show that this technique enables sequential filling of the molten metal, effectively eliminating shrinkage porosity defects in the thick-walled areas. Wang et al. [18] established an alloy design method that integrates computational thermodynamics and machine learning, successfully predicting the phase transformation behavior of Al-Si-Mg-Sc alloys. Zhao et al. [19] used the MAGMAsoft software to perform numerical simulations of the filling and solidification processes of AlSi9Cu3 alloy in the production of transmission housings. They found that the fan-shaped gating system causes significant shrinkage and porosity defects in the parts. By incorporating a cooling system and strategically controlling the occurrence of shrinkage and porosity defects in the overflow regions, these defects can be effectively eliminated during subsequent machining, significantly improving the overall quality of the product.

However, a few studies have investigated the microstructure evolution and mechanical properties of the alloy with different filling distances and thicknesses based on simulation-assisted manufacturing. In this paper, the mechanical properties and microstructure evolution of the alloy with different filling distances and thicknesses were investigated. The findings of this study provide theoretical guidance for the integrated forming of large structural components in new energy vehicles and contribute to the development of high-strength ductile die-casting aluminum alloy material systems.

## 2. Experimental Procedures

### 2.1. Materials Preparation

**Figure 1:**
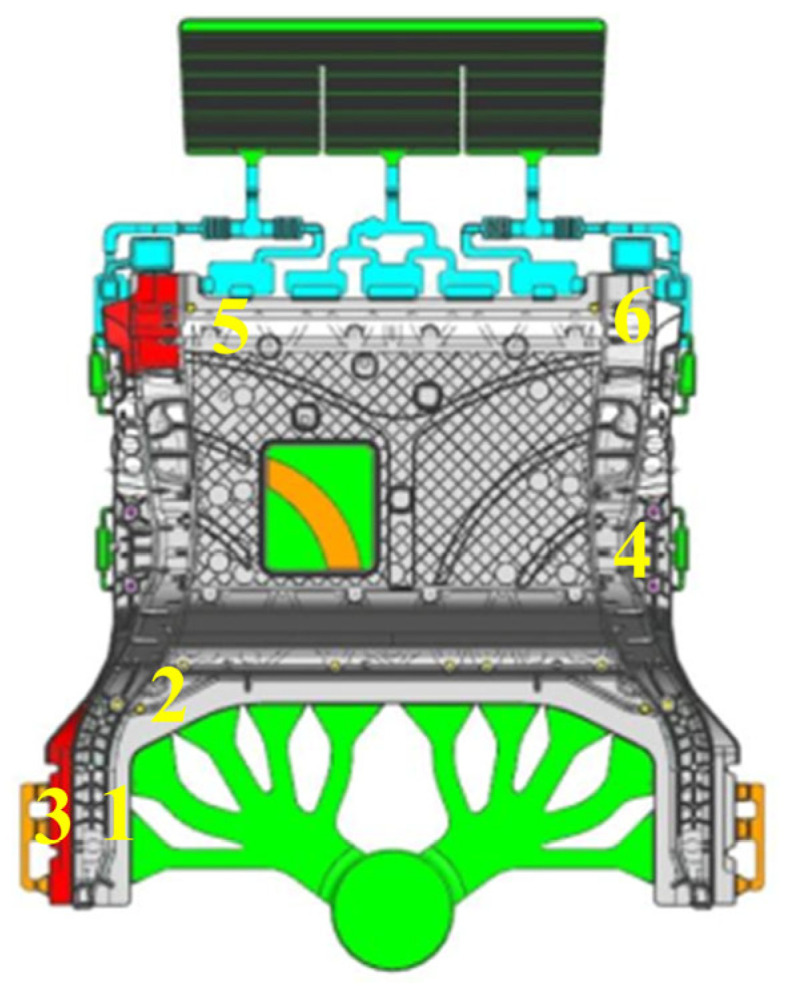
A 3D model of the automotive rear floor and sample locations for tensile testing.

The casting material used was the MRL-1 non-heat-treatment die-casting aluminum alloy, developedby Chongqing Changan Automobile Co., Ltd., Lihuan, China with its specific composition provided in Table 1. A 3D model of an automotive rear floor was created using the CATIA software (CATIA V5 R21). The casting filling process was simulated using the Zhizhu Super Cloud software (SupreCAST V2.0) developed by Beijing Shichuang, China. The simulation parameters are detailed in Table 2. In this study, the effect of material viscosity on the filling process was considered in the simulation. The temperature-dependent viscosity data for the alloy are provided in Figure S1. Based on the structural characteristics of the die-cast component, the relatively flat bottom of the rear floor was selected as the parting surface for mold design. The die-casting mold was fabricated using H13 die steel, with each mold producing a single component. The castings were produced using the LEAP 7000T die-casting machine by Yizumi, Foshan, China. The pouring temperature of the molten aluminum was maintained at 700 °C, with the mold temperature ranging from 120 °C to 200 °C. The injection speed was set to 5 m/s, and the vacuum level was controlled at 50 mbar. The maximum contour dimensions of the rear floor were 1500 mm × 1200 mm × 650 mm, with a single-piece weight of 51 kg. The casting had a complex structure with uneven thickness, including multiple irregular bosses, reinforcing ribs, and several threaded holes distributed around its bottom. Samples were taken from the die-cast components for testing the mechanical properties and for microstructural observation, with the sampling locations indicated in Figure 1. The filling distance of different samples were as follows: #1–2: 200 mm; #3: 210 mm; #4: 450 mm; and #5–6: 1300 mm. The thicknesses of the different samples were as follows: Sample #1#: 4.10 mm; Sample #2#: 5.30 mm; Sample #3#: 4.14 mm; Sample #4#: 3.36 mm; Sample #5#: 2.94 mm; and Sample #6#: 4.93 mm.

### 2.2. Microstructural Characterization

**Figure 2:**
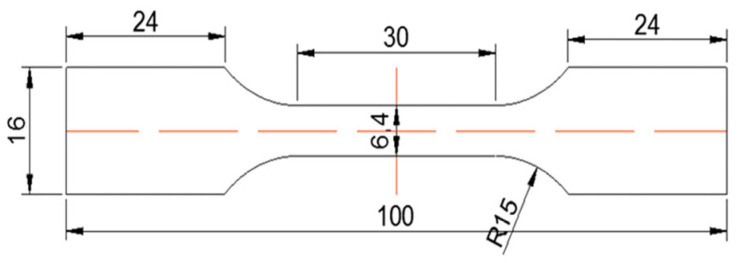
Diagram of the dimensions of the tensile specimens.

The dimensions of the tensile specimens are shown in Figure 2. Tensile tests were conducted at room temperature using a WDW300 electronic universal testing machine, Jinan, China, at a strain rate of 1 mm/min, with three parallel specimens tested. Samples for microstructural observation were taken from the same locations as the tensile specimens. The microstructure of the alloy was examined using a Leica metallographic microscope (DMI5000M, Bensheim, Germany), with Keller’s reagent used as the etchant. The composition of Keller’s reagent is 1 mL of HF + 1.5 mL of HCl + 2.5 mL of HNO_3_ + 95 mL of H_2_O, and the etching time was from 5 to 10 s. For further detailed examinations of the morphology and distribution of second-phase particles in the microstructure, as well as the fracture surface morphology, scanning electron microscopy (SEM, JCM-7000 produced by Japan Electronics Co., Ltd., Tokyo, Japan) equipped with energy dispersive spectroscopy (EDS) was used. The phase composition of the alloy was identified using a Panalytical X-ray diffraction instrument (Malvern Panalytical Ltd., Malvern, UK). The diffraction was conducted using Cu-Kα radiation with a diffraction angle range of 20° to 90°.

## 3. Results

### 3.1. Filling Process

**Figure 3:**
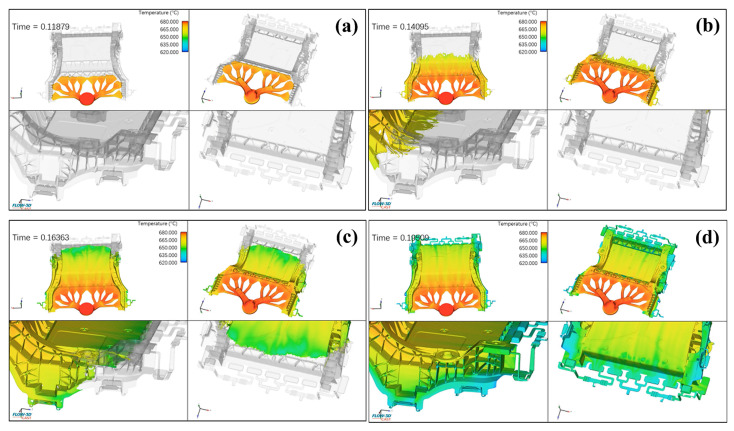
Simulation results of the filling process. (**a**) t = 0.11879 s; (**b**) t = 0.14095 s; (**c**) t = 0.16363 s; (**d**) t = 0.19509 s.

Figure 3 presents the simulation results of the filling sequence in the fan-shaped gating system. The corresponding simulation video is provided in Supplementary Materials: (Video S1: Simulation of the filling process; Video S2: Simulation of the filling sequence; Video S3: Simulation of the cooling rate). Molten metal is injected from the chamber into the die, with a total filling time of 0.195 s. The filling process is divided into two stages: low-speed filling and high-speed filling. In the low-speed filling stage, as shown in Figure 3a, at t = 0.11879 s, molten metal is injected synchronously from the horizontal runner to the inner gate along the deep cavity side in a fan-shaped pattern. This ensures the complete expulsion of air from the gating system, thereby reducing the formation of gas-related defects. In the high-speed filling stage, at t = 0.14095 s, the molten metal reaches the bottom and extends upward in a linear pattern, as shown in Figure 3b. At the locations of the bosses and locating pins, the uneven depth of the surrounding areas causes turbulence in the molten metal. However, overflow channels were pre-installed at these locations to efficiently remove oxide inclusions and gases. At t = 0.16363 s, the molten metal reaches the edge of the casting, and filling of the entire cavity is essentially complete. By t = 0.19509 s, the aluminum melt successfully and smoothly completed the filling process, with the liquid surface rising steadily. The pre-installed overflow channels effectively prevented the occurrence of gas-related defects. These channels are the last areas to be filled with molten metal, ensuring complete expulsion of gases during the filling process. The simulation results also indicate that the alloy melt temperature decreases as the distance from the gate increases. The temperature difference between the gate and the filling end positions is approximately 60 °C.

### 3.2. Microstructure Evolution

**Figure 4:**
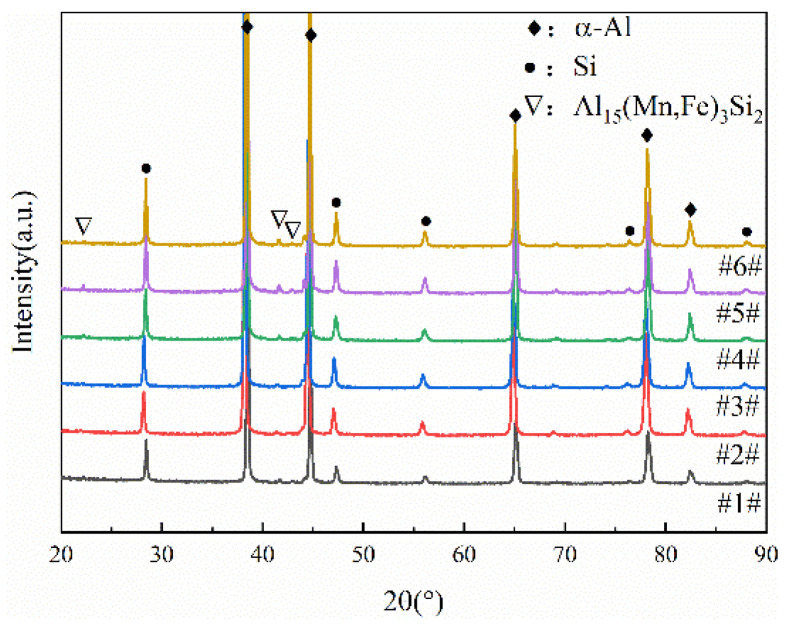
XRD patterns of the alloy at different sampling locations (Samples #1–6# correspond to the locations labeled 1–6 in Figure 1).

Figure 4 presents the X-ray diffraction (XRD) spectra of samples taken from different locations of the product. All samples consist of α-Al, Si and the Al_15_(Mn, Fe)_3_Si_2_ intermetallic phases [20,21,22]. Due to the relatively low Fe content in the alloy, the diffraction peak intensity corresponding to the Al_15_(Mn, Fe)_3_Si_2_ intermetallic phase is lower compared to the main peak of α-Al. Moreover, the positions and intensities of the diffraction peaks exhibit minimal variation across the different filling distances and thicknesses. This suggests that filling distance and thickness have a negligible impact on the type of second phase in the alloy microstructure.

**Figure 5:**
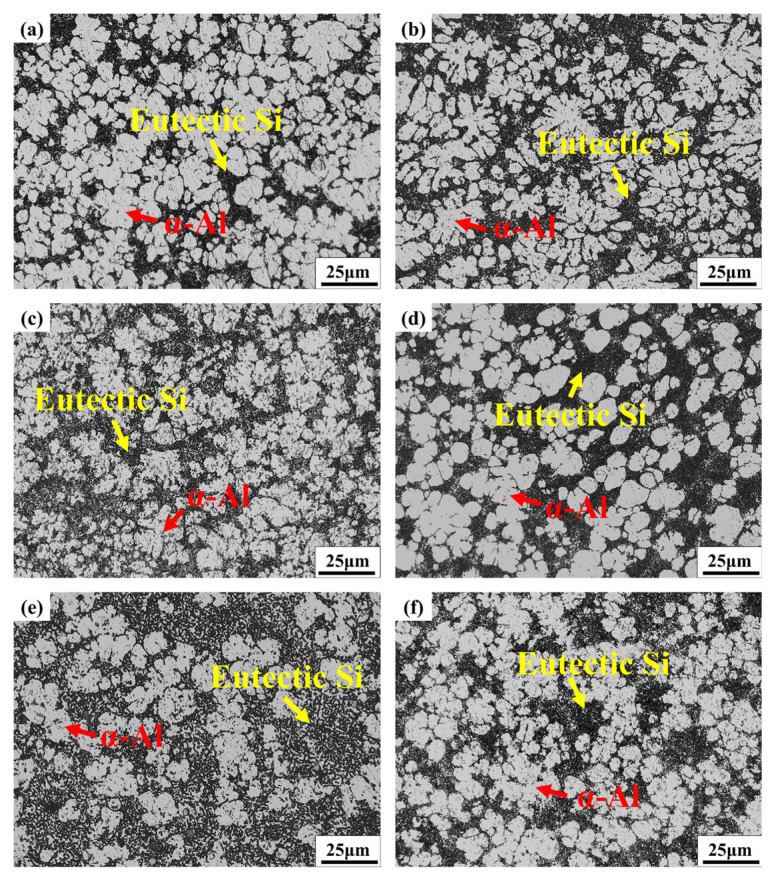
OM microstructure of samples from positions #1–6 ((**a**): #1; (**b**): #2; (**c**): #3; (**d**): #4; (**e**): #5; (**f**): #6).

Figure 5 presents the optical microscopy (OM) microstructure images of samples from positions #1–6. The microstructure of the samples primarily consists of primary α-Al dendrites, with eutectic structures (α-Al+ Si) between the dendrites and dark gray intermetallic compounds localized near the eutectic Si [23]. Significant differences in microstructure are observed across the samples taken from different locations. A detailed analysis, combining simulation results with the specific sampling locations, is required. Although the filling distances of Samples #1# and #2# are identical, their metallographic structures exhibit significant differences. An analysis of the filling-process simulation results, shown in Figure 3, reveals that both Samples #1 and #2 are preferentially filled due to their proximity to the gate. As the filling process progresses, the simulation indicates a minimal difference in the liquid metal temperature between the two locations. This suggests that the disparity in alloy microstructure between Samples #1 and #2 may be attributed to differences in thickness. The underlying causes will be analyzed in detail below. Samples from positions #3 and #4 were taken from regions close to and far from the gate, respectively. The simulation results indicate that at position #3, where the filling distance is 210 mm, the temperature of the molten metal is relatively high during filling, providing sufficient time for nucleation and grain growth. Consequently, the α-Al grains in the microstructure of Sample #3 tend to form dendritic structures. In contrast, at position #4, where the filling distance increases to 450 mm, the temperature of the molten metal decreases to some extent as it passes through this region. The shorter solidification time suppresses the nucleation and growth of α-Al grains, resulting in a predominantly spherical grain structure. The filling distance at both positions #5 and #6 is 1300 mm. Microstructural observations indicate that the α-Al morphology at these two positions shows minimal differences. Therefore, it can be concluded that as the filling distance increases, the temperature of the molten metal decreases, causing the α-Al grains in the alloy microstructure to transition from a petal-like morphology to a coexistence of both petal-like and spherical grains.

**Figure 6:**
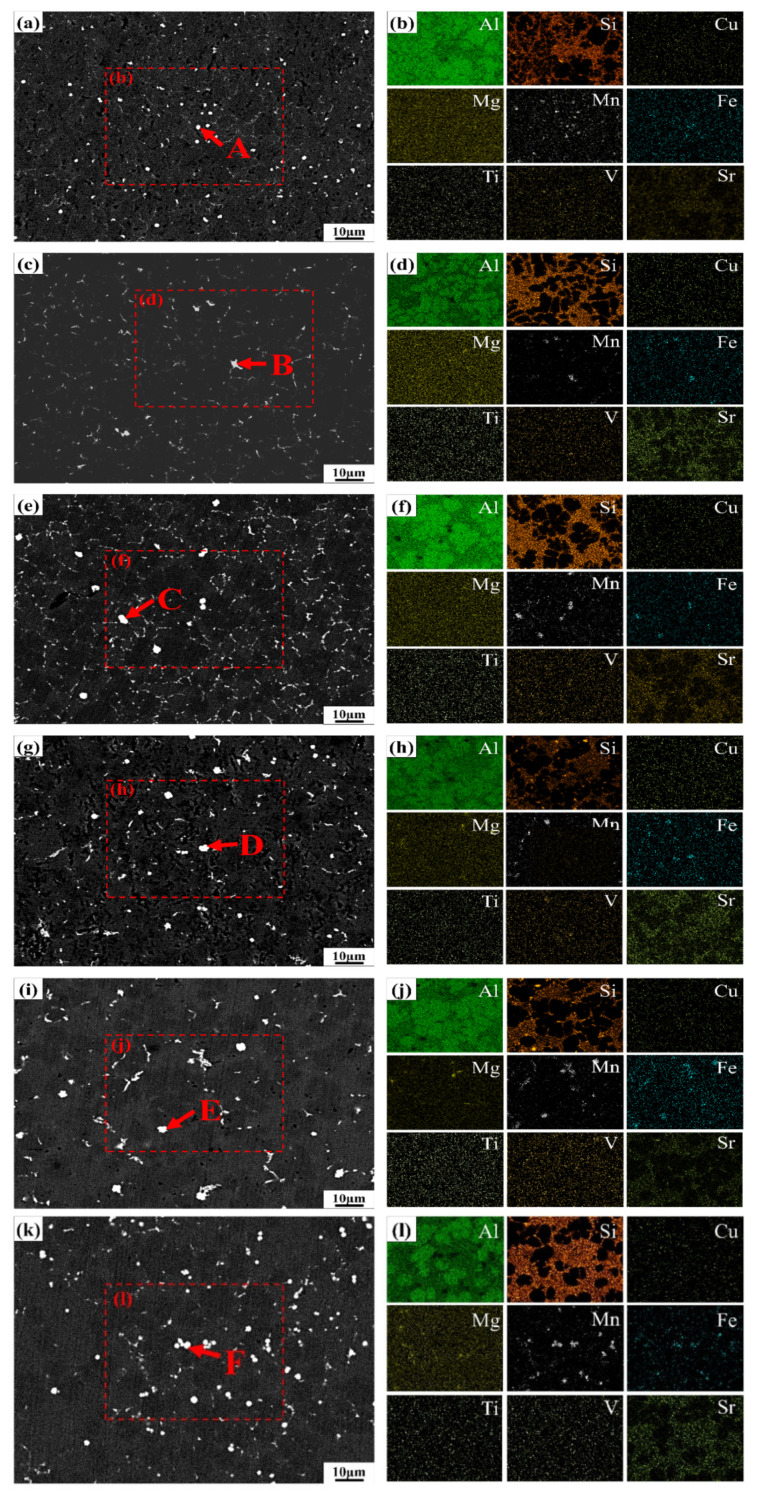
Second phase morphology and EDS results of alloys at different locations ((**a**,**b**): #1; (**c**,**d**): #2; (**e**,**f**): #3; (**g**,**h**): #4; (**i**,**j**): #5; (**k**,**l**): #6).

To facilitate a comprehensive analysis of phase distribution and morphological characteristics in the microstructure at different sampling locations, scanning electron microscopy (SEM) and energy-dispersive spectroscopy (EDS) analyses were performed on samples from various positions. Figure 6 presents the SEM and EDS analysis results at different sampling locations. Combined with optical microscopy (OM) microstructure images, X-ray diffraction (XRD) patterns, and EDS area scan results, it can be concluded that the alloy microstructure consists of an α-Al matrix (black), eutectic Si (gray), and intermetallic compounds (bright white). The α-Al dendrites primarily exhibit a petal-like morphology, while the α-Al–Si eutectic is distributed as granular or short rod-like structures within the regions between primary α-Al dendrites. The white particulate intermetallic compounds are mainly located along the grain boundaries. According to the EDS analysis results, shown in Table 3, the intermetallic compounds are primarily identified as an Al_15_(Mn, Fe)_3_Si_2_ (α-Fe) phase, composed of Al, Si, Mn, and Fe elements. Vanadium (V) elements may replace Fe atoms in the α-Fe phase, which explains the detection of V in the intermetallic compounds [24]. Although Mg exhibited localized segregation in certain regions, no Mg-containing secondary phases were detected in the alloy due to the low overall Mg content. The distribution of Sr atoms was similar to that of Si atoms, with Sr primarily serving to modify the eutectic Si structure within the alloy [25,26]. Although Samples #1 and #2 had the same filling distance of 200 mm, the α-Fe phase in Sample #1 exhibited a uniformly spherical morphology, whereas in Sample #2, partial segregation of α-Fe was observed, forming irregular block-like structures dispersed within the matrix. As the filling distance increased, the degree of the α-Fe phase aggregation also increased, appearing either as clustered particles or as irregular blocks distributed throughout the matrix. At locations with longer filling distances, the lower metal temperature and longer filling distance increase the proportion of intermetallic compounds and the likelihood of segregation and coarsening. In conclusion, as the filling distance increases, the proportion of second-phase intermetallic compounds in the alloy increases, along with a greater likelihood of their agglomeration and coarsening.

### 3.3. Mechanical Property Analysis

**Figure 7:**
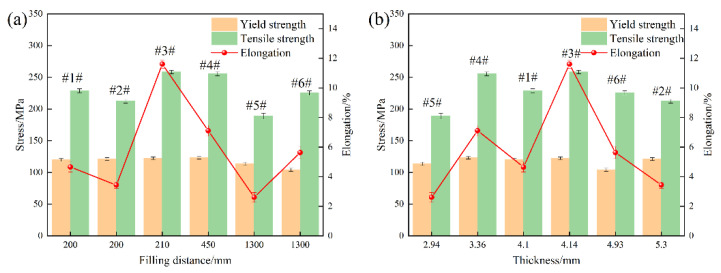
Variation of mechanical properties with filling distance and thickness: (**a**) Variation of mechanical properties with filling distance; (**b**) Variation of mechanical properties with thickness.

To investigate the variation in mechanical properties at different sampling positions during the casting process, mechanical testing was conducted on samples taken from the positions shown in Figure 1. As the filling distance or thickness increases, the changes in mechanical properties are illustrated in Figure 7. The results indicate that the optimal filling distance of the alloy ranges from 210 to 450 mm, while the optimal thicknesses range from 3.36 to 4.14 mm. With the filling distance and thickness increase, the yield strength, tensile strength, and elongation of the alloy initially increase and then decrease. The optimal properties are achieved when the filling distance is 210 mm and the thickness is 4.14 mm, with a yield strength of 122.35 MPa, tensile strength of 258.43 MPa, and elongation of 11.60%. At the same filling distance, near the gate position, when the thickness increases from 4.1 mm to 5.3 mm, the alloy’s tensile strength and elongation decrease. However, at positions farther from the gate, when the thickness increases from 2.94 mm to 4.93 mm, both the tensile strength and elongation of the alloy increase. Although Samples #1 and #2 had the same filling distance, their mechanical properties differed significantly. According to the filling simulation results, both samples completed filling early due to their proximity to the gate. During the subsequent filling of the remaining regions, excess molten metal flowed through adjacent overflow channels. However, due to its greater thickness, Sample #2 exhibited more pronounced microstructural evolution. Specifically, microstructural observations revealed well-developed α-Al dendrites and localized aggregation of blocky α-Fe phases in Sample #2, indicating slower solidification. The prolonged solidification allowed more time for the nucleation and growth of secondary phases. As a result, the coarse microstructure exhibited poor deformation compatibility during tensile loading, leading to a lower yield strength, ultimate tensile strength, and elongation compared to Sample #1. Samples #3 and #4 had filling distances of 210 mm and 450 mm, respectively. Under high-speed, high-pressure conditions, casting defects were pushed toward the flow front during filling. Although Sample #3 had a relatively large thickness, its shorter filling distance reduced defect accumulation, and SEM analysis confirmed minimal intermetallic segregation. In contrast, Sample #4, with a longer filling distance, experienced lower metal temperatures and slower flow velocities during the final stages of filling. As a result, impurities were not effectively expelled through the overflow system, making the filling distance a more critical factor affecting mechanical performance. Consequently, Sample #4 showed a lower yield strength, tensile strength, and elongation than Sample #3, although its overall mechanical properties remained superior to those of Samples #1 and #2. Samples #5 and #6 had a filling distance of 1300 mm. As the thickness increased from 2.94 mm to 4.93 mm, both tensile strength and elongation improved. In conclusion, the coupled effect of thickness and filling distance is the primary factor influencing the variation in mechanical properties. This overall trend suggests that with an increasing thickness and filling distance, yield strength, tensile strength, and elongation initially increase and then decrease. The optimal filling distance was found to be between 210 mm and 450 mm, and the optimal thickness ranged from 3.36 mm to 4.14 mm.

**Figure 8:**
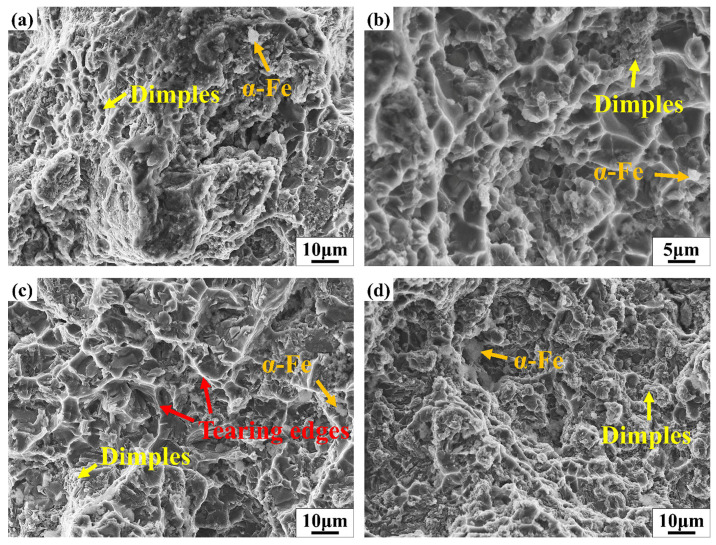

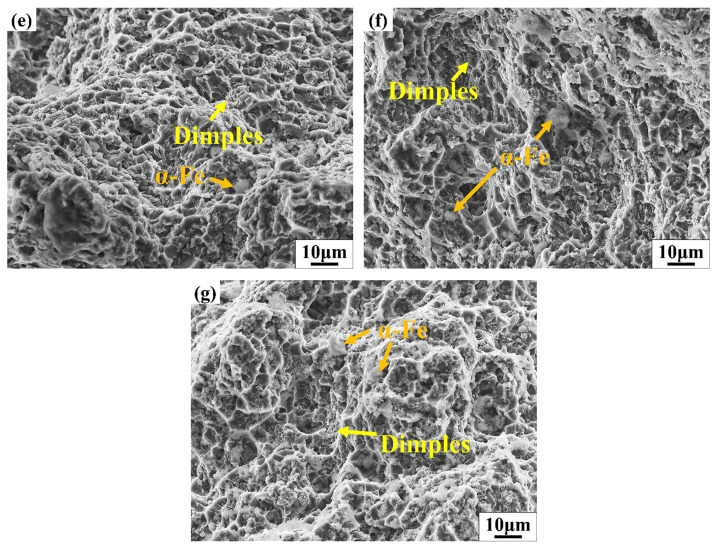
Tensile fracture morphology of samples at different sampling locations ((**a**): #1; (**b**): magnified morphological image of Sample #1; (**c**): #2; (**d**): #3; (**e**): #4; (**f**): #5; (**g**): #6).

Figure 8 shows the tensile fracture morphology of samples from different sampling locations. The alloy exhibits ductile fracture behavior, with fracture surfaces primarily composed of dimples and a small number of α-Fe [27,28]. Figure 8a,b shows the tensile fracture morphology and corresponding magnified image of Sample #1#. As observed in the magnified view, a large number of small and densely distributed dimples—approximately 1 μm in size—are present on the fracture surface, which is indicative of a ductile fracture mode. Figure 8c shows the tensile fracture morphology of Sample #2#. Due to the coarser dendritic α-Al structure in Sample #2, its fracture surface exhibits fewer dimples and more prominent tearing ridges compared to Sample #1, indicating lower ductility. Figure 8d,e shows the fracture morphologies of Samples #3 and #4, both of which display finer dimples on the fracture surfaces, suggesting better plasticity. Figure 8f,g illustrates the tensile fracture morphologies of Samples #5 and #6. Owing to the longer filling distances and the observed segregation of α-Fe phases in SEM images, large blocky α-Fe phases are evident on the fracture surfaces of Samples #5 and #6. The extended filling distance, reduced molten metal temperature, and increased presence of bright intermetallic compounds contributed to the deterioration of the material’s mechanical properties.

## 4. Conclusions

This study employed simulation-assisted manufacturing to investigate the spatial heterogeneity of microstructure and mechanical properties in large die-cast components. It offers valuable guidance for optimizing integrated die-casting process parameters and material design, providing practical engineering insights for advancing lightweight manufacturing technologies in new energy vehicles. The key conclusions of this study are as follows:

(1) The alloy microstructure consists of α-Al, an Al-Si eutectic, and the Al_15_(Mn,Fe)_3_Si_2_ intermetallic phases. Near the gate, the higher molten metal temperature promotes the formation of well-developed petal-like α-Al dendrites. In contrast, the increased cooling rate in the distant thin-walled regions facilitates the formation of equiaxed grains. With an increasing filling distance, the Al_15_(Mn,Fe)_3_Si_2_ phase tends to segregate and coarsen.
(2) The coupled effect of thickness and filling distance is the primary factor influencing the variation in mechanical properties. The optimal filling distance of the alloy ranges from 210 mm to 450 mm, while the optimal thickness ranges from 3.36 mm to 4.14 mm.
(3) With a filling distance and thickness increase, the yield strength, tensile strength, and elongation of the alloy initially increase and then decrease. The optimal properties are achieved when the filling distance is 210 mm and the thickness is 4.14 mm, with a yield strength of 122.35 MPa, tensile strength of 258.43 MPa, and elongation of 11.60%.
(4) At the same filling distance, near the gate position, when the thickness increases from 4.1 mm to 5.3 mm, the alloy’s tensile strength and elongation decrease. However, at positions farther from the gate, when the thickness increases from 2.94 mm to 4.93 mm, both the tensile strength and elongation of the alloy increase.

## Figures and Tables

**Table 1:** The chemical composition of the MRL-1 alloy (wt. %).

Si	Mg	Mn	Fe	Zn	Ti	V	Sr	Al
9.0	0.20	0.50	0.10	0.05	0.12	0.12	0.02	Bal.

**Table 2:** Parameters used in the filling simulation of castings.

Parameters	Value
Diameter of the pressure chamber	270 mm
Effective length of the pressure chamber	1660 mm
Thickness of the charge cake	40 mm
Low-speed phase of the plunger	0.8 m/s
High-speed phase of the plunger	5 m/s
Pouring temperature	700 °C
Vacuum level	50 mbar
Mold temperature	200 °C

**Table 3:** EDS analysis results of intermetallic compounds in Figure 6 (at.%).

Point	Elements				
	Al	Si	Mn	Fe	V
A	77.8	13.35	7.48	1.37	0
B	71.22	12.70	12.41	2.25	1.42
C	69.48	13.67	13.11	2.32	1.42
D	69.76	13.28	13.13	2.43	1.40
E	70.86	13.56	12.08	2.28	1.22
F	70.72	13.80	11.93	2.32	1.23

## Data Availability

The raw/processed data required to reproduce these findings cannot be shared at this time, as the data also form part of an ongoing study.

## Supplementary Materials

The following supporting information can be downloaded at: https://www.mdpi.com/article/10.3390/ma18092143/s1, Figure S1: The temperature-dependent curves of thermodynamic and dynamic viscosity for the alloy material; Video S1: Simulation of the filling process; Video S2: Filling sequence; Video S3: Cooling rate.
